# Gut microbiota shift in layer pullets fed on black soldier fly larvae-based feeds towards enhancing healthy gut microbial community

**DOI:** 10.1038/s41598-022-20736-0

**Published:** 2022-10-06

**Authors:** Evalyne W. Ndotono, Fathiya M. Khamis, Joel L. Bargul, Chrysantus M. Tanga

**Affiliations:** 1 International Centre of Insect Physiology and Ecology (ICIPE), Nairobi, Kenya; 2 Department of Biochemistry, Jomo Kenyatta University of Agriculture and Technology (JKUAT), Nairobi, Kenya

**Keywords:** Microbiology, Molecular biology

## Abstract

Globally, most gut microbiota-related studies have focused on broilers due to their diverse microbial communities compared to that of layer chicken. However, in Africa few studies have been undertaken despite the increasing benefits to the poultry industry. The utilization of Insect-Based diets to improve the gastrointestinal function and gut health in poultry is increasingly gaining global attention. Here, we evaluated the potential roles of commercial black soldier fly larvae-based feeds (BSFLF) in reshaping the abundance, composition and diversity of the gut microbiota of layer chickens using high throughput Oxford nanopore Minion sequencing of the full length bacterial 16S rRNA gene. Two hundred and fifty ISA Brown layer chicks were reared in pens for a period of 20 weeks. The layer pullets were allotted 5 dietary treatments that were formulated as follows: control diet (T1): 100% FM + 0% BSFL, T2: 25% BSFL + 75% FM; T3: 50% BSFL + 50% FM; T4: 75% BSFL + 25% FM, and T5: 100% BSFL + 0% FM. Sampling was done from the eight major regions including oesophagus, crop, proventriculus, gizzard, duodenum, ileum, large intestines and ceca. Out of the 400,064 classified reads analyzed, the most dominant phyla identified across the feed treatments were Firmicutes, Bacteroidetes, Proteobacteria and Actinobacteria. The diet treatment with 100% inclusion levels of BSFL showed the highest intra-species alpha diversity and richness according to Chao1 and Shannon index. Intra-species beta diversity assessment revealed that the diet types significantly influenced the abundance of the microbiota, but differences between most abundant taxa were similar. There was increase in abundance of potentially beneficial bacteria (*Lactobacillus*, *Bacteroides* and *Enterococcus*) with increased inclusion levels of BSFLF in layer pullets diets. Across the different gut segments, *Lactobacillus* dominated all the eight regions and the ceca was the most diverse segment. Our findings unravel complex gut microbial shift in laying hen fed BSFLF and therefore underpins the potential roles of beneficial bacteria as promising prebiotics and probiotics in reshaping of the gut microbiota to maintain good gut health.

## Introduction

Insects have proven to be a nutrient—rich ingredient in animal feed, and its sustainable use is gaining momentum worldwide due to the rising costs of major protein sources such as fish meal, soybean, cotton seed cake, among others, which are used in animal feeds^1^. The rapidly increasing human population growth is anticipated to lead to increased demand for animal proteins^2,3^. In Sub-Saharan Africa, domestic chicken production forms the basis of the protein industry with more than 80% of the smallholder farmers contributing significantly to the growth of the poultry industry. But one of the major constrain hampering this industry from attaining its full production potential has been the availability and accessibility to nutritious protein rich feeds^4,5^.

Protein is an essential key ingredient in poultry feeds as it is necessary for their growth, body maintenance and high carcass quality^6,7^. Protein alone accounts for over 70% of the total cost of livestock production and lack of such protein supplements continues to limit efficient poultry production^6^. In conventional farming systems, soybean meal and fishmeal are the main sources of protein in poultry feeds^8,9^ This has led to an increased demand for the soybean and fishmeal-based commercial feeds which has caused the availability of soybean and fishmeal to be limited due to the high cost prices and scarcity of resources^10^. Soybean and fishmeal supply has also been limited due to the factor that they are also used for human consumption hence creating an imbalance between the demand and supply^11^. Therefore, to meet the nutritive requirements for poultry production, there is need to identify low cost, locally available, and sustainable protein sources for animal feeds.

The black soldier fly (BSF), *Hermetia illucens* L. (Diptera: Stratiomyidae), is one such promising insect. It is commonly reared for industrial purposes and its peculiar benefits such as bioremediation of organic wastes due to their ability to convert organic waste substrates into biomass containing high quality crude protein ranging between 35–57%, better amino acid profiles, high growth rates and little space is required for rearing^12–15^*.* Since BSF feeds on a variety of organic wastes, it is highly probable that they could acquire microorganisms (either beneficial or harmful) from these substrates, which may be transferred to animal feeds^16–18^*.* Interactions between the gut microbiota and the chicken plays a key role in host development, health and nutrition, and food safety^19,20^. The microbial communities of layer chicken gut has been shown to play vital roles in immunity, physiological performance, and nutrition development, thus any changes in their gut microbiome may have adverse effects on productivity, feed efficiency, and overall health^20–22^. Therefore, chicken diet is thought to be the main determinant influencing the composition of the gut microbiota and thus changes in feed components and additives would contribute to variation in species composition and diversity of the gut microbiome^23^.

Presently, extensive research has been devoted to the nutritional value of the BSF and optimization of rearing conditions, overlooking the impact of pathogenic species and contaminants in animal nutrition. According to^24^, insects for feed are usually processed with their intestinal contents that can harbor various transmissible microorganisms whose microbiota can also be expressed on the exoskeleton. Hence, insect meal diet formulations are a crucial aspect whereby safety precautions must be undertaken to ensure safety of insect-based feeds. The working hypothesis of this study is that the analysis of the microbial communities could lead to the identification of species with unique characteristics that can be isolated and exploited for further research. Other than playing an important role in its host by providing proteins, the BSF larvae inclusion in the diet could have an improved effect on the gut and overall health of the chicken^25^*.* The aim of this study was to evaluate the bacterial diversities present in the gut of the layer pullets after being fed diets with various inclusion levels of black soldier fly larvae (BSFL) meal and also to assess microbial communities between the different gut sections.

## Materials and methods

### Ethical statement

All experiments were conducted with strict adherence to the approved experimental guidelines and procedures by the Institutional Animal Care and Use Committee (IACUC) at the Kenya Agricultural and Livestock Research Organization (KALRO)—Veterinary Science Research Institute (VSRI); approval Code No.: KALRO—VSRI/IACUC019/30082019. We confirm that all experiments were performed in compliance with the ARRIVE guidelines (https://arriveguidelines.org).

### Chicken rearing and diet formulations

The chickens were reared at the Poultry Research Unit in KALRO located in Naivasha, Nakuru County, Kenya (Lat 0.6835° S, longitude 36.4012° E) as described by^26^. A total of 250 one day old female ISA Brown layer chicks were sourced from Kenchic Limited, Nairobi. They were all kept in a brooder and for a period of 14 days, the young birds were provided the control (100% FM inclusion ratio) diet and water ad libitum. After the 14 days, the birds were kept in pens each with 5 chickens and allotted randomly to one of the five dietary treatments. Each experimental setup was replicated 9 times. They were reared for 20 weeks and the birds were given access to both feed and clean water ad libitum daily. The birds were all vaccinated following a program as shown in Table S1. All ingredients required for the formulation of the various diet types for both the chicks (Table 1) and the growers (Table 2) were sourced and formulated as described by^26^. The BSF larvae were obtained from the International Centre of Insect Physiology and Ecology (icipe), Nairobi, Kenya. The larvae were harvested at the 5th instar, oven-dried at 120 °C using a hot air circulating drying oven (Henan Forchen Machinery, Luoyang, Henan, China) and ground into powder which was mixed with the other raw materials for diet formulation. A standard diet of commercialized conventional fishmeal (FM) served as the control diet (T1): 100% FM + 0% BSFL, while the other 4 diets were composed of: (T2): 25% BSFL + 75% FM; T3: 50% BSFL + 50% FM; T4: 75% BSFL + 25% FM, and T5: 100% BSFL + 0% FM. The hens were reared under appropriate room conditions with good lighting, temperature kept at 30 ± 1 °C and humidity at 70 ± 2% following protocols and guidelines as instructed by the Federation of Animal Science Societies^27^ until the hens were ready for sample collection.

**Table 1:** Feed composition of the formulated diets fed to chicks for a period of eight (8) weeks^26^.

Diet types					
Ingredients (%)	Diet 1 (control)	Diet 2	Diet 3	Diet 4	Diet 5
Maize germ	60.0	60.0	60.0	60.0	60.0
Soybean meal	21.0	21.0	21.0	21.0	21.0
Fishmeal	10.0	7.5	5.0	2.5	0.0
Black soldier fly larvae	0.0	2.5	5.0	7.5	10.0
Vegetable oil	2.0	2.0	2.0	2.0	2.0
Limestone	5.0	5.0	5.0	5.0	5.0
Dicalcium phosphate	1.5	1.5	1.5	1.5	1.5
Iodized salt (NaCl)	0.3	0.3	0.3	0.3	0.3
Super Layer premix	0.2	0.2	0.2	0.2	0.2

Super layer premix contents per 2.5 kg: Vit. (Vitamin) A: 8,000,000 IU/kg, Vit. D3:2,000,000 IU/kg, Vit. E: 3000 mg, Vit. K3: 2000 mg, Vit B2: 3500 mg, Pantothenic Acid: 6600 mg, Niacin: 20,000 mg, Folic Acid: 550 mg, Vit. B12: 6 mg, Choline chloride: 200,000 mg, Lysine: 350 mg, Methionine: 120 mg, Manganese: 63,000 mg, Iron: 23,000 mg, zinc: 63,000 mg, Copper: 14,000 mg, Cobalt: 1000 mg, Iodine: 2000 mg, Selenium: 100 mg and BHT: 120,000 mg. Abbreviation: BSFL- black soldier fly larvae. FM- fishmeal Diet 1—0% BSFL, Diet 2—25% BSFL and 75% FM, Diet 3—50% BSFL and 50% FM, Diet 4—75% BSFL and 25% FM and Diet 5—100% BSFL.

**Table 2:** Feed composition of the formulated diets fed to grower layer chicken for a period of twelve (12) weeks^26^.

Diet types					
Ingredients (%)	Diet 1 (control)	Diet 2	Diet 3	Diet 4	Diet 5
Maize germ	50.0	50.0	50.0	50.0	50.0
Pollard (wheat)	19.0	19.0	19.0	19.0	19.0
Soybean meal	13.0	13.0	13.0	13.0	13.0
Fishmeal	10.0	7.5	5.0	2.5	0.0
Black soldier fly larvae	0.0	2.5	5.5	7.5	10.0
Limestone	5.0	5.0	5.0	5.0	5.0
Dicalcium phosphate	2.0	2.0	2.0	2.0	2.0
Iodized salt (NaCl)	0.3	0.3	0.3	0.3	0.3
Layer premix	0.2	0.2	0.2	0.2	0.2

Super layer premix contents per 2.5 kg: Vit. (Vitamin) A: 8,000,000 IU/kg, Vit. D3:2,000,000 IU/kg, Vit. E: 3000 mg, Vit. K3: 2000 mg, Vit B2: 3500 mg, Pantothenic Acid: 6600 mg, Niacin: 20,000 mg, Folic Acid: 550 mg, Vit. B12: 6 mg, Choline chloride: 200,000 mg, Lysine: 350 mg, Methionine: 120 mg, Manganese: 63,000 mg, Iron: 23,000 mg, zinc: 63,000 mg, Copper: 14,000 mg, Cobalt: 1000 mg, Iodine: 2000 mg, Selenium: 100 mg and BHT: 120,000 mg. Abbreviation: BSFL- black soldier fly larvae. FM- fishmeal Diet 1—0% BSFL, Diet 2—25% BSFL and 75% FM, Diet 3—50% BSFL and 50% FM, Diet 4–75% BSFL and 25% FM and Diet 5—100% BSFL.

### Sample collection

At 20 weeks old, a subset of 75 chickens was selected randomly with 15 chickens per treatment being used for sample collection. The chickens were slaughtered professionally and the whole gut was aseptically harvested from each chicken. All efforts were made to minimize pain, stress, and discomfort in the animals. Samples were excised from the 8 major sections of the gut including the esophagus, crop, proventiculus, gizzard, duodenum, ileum, large intestine and the ceca. The samples were then placed in clean Eppendorf tubes and stored at 4 °C on ice for transportation to the Arthropod Pathology Unit at icipe, Kenya for further analysis.

### Genomic DNA extraction and 16S rRNA amplification

Gut contents were sampled from the inner gut epithelial tissues and used for genomic DNA extraction using Isolate II Genomic Extraction kit (Bioline, London, UK) following the manufacturer’s instructions. The concentration and quality of DNA was determined using a nanodrop 2000/2000c spectrophotometer (Thermo Fischer Scientific, Wilmington, USA) and those with good quality range of 1.8–2.0, based on A_260nm_/A_280nm_ were selected for 16S metagenomics analyses. Sequencing of the full length ~ 1500 bp bacterial 16S rRNA gene was performed using Oxford Nanopore Technologies (ONT) Minion device using R9.4.1 flow cells and the libraries were prepared with pooled DNA for multiplexing using the 16S barcoding kit SQK-16S024. Library preparation with PCR step was conducted using the following components: 10 pmol μL^−1^ of each 16S barcode, 10 ng μL^−1^ of DNA template, 0.625 U μL^−1^ *MyTaq* DNA polymerase (Bioline), and 5 × *MyTaq* reaction buffer (5 mM dNTPs, 15 mM MgCl_2_, stabilizer and enhancer) (Bioline). The reactions were set up in a total reaction volume of 50 µL and run in a Master cycler Nexus gradient thermal cycler (Eppendorf, Germany), under the following conditions: initial denaturation for 2 min at 95 °C, followed by 35 cycles of denaturation for 30 s at 95 °C, annealing for 40 s at 55 °C, extension for 1 min at 72 °C, and a final extension step of 10 min at 72 °C. The libraries were then purified using a Bioline kit following the manufacturer’s instructions and pooled together prior to loading into the flow cells for Minion sequencing.

### Sequencing and data analysis

The sequencing runs were done for 4 h with live base calling performed using the Albacore tool (v2.3.4) on the Minknow (v 20.10.3) software on the ONT cloud. The sequencing runs generated FASTQ files which were uploaded to EPI2ME software v 2020.11.19 (https://epi2me.nanoporetech.com) for qualitative, real—time species identification from metagenomic samples. The FASTQ—16S workflow (v 2020.04.06) on EPI2ME was used to characterize the reads and assign taxonomy to the genus level using NCBI as the reference database and relative cumulative abundance plots were generated. A minimum abundance threshold of 0.5% was used to select the most abundant taxa and those that were below this threshold were collapsed into others. However, EPI2ME is a limited tool since it does not give the diversity parameters such as alpha and beta diversity. Thus, QIIME2—v 2020.8^28^ pipeline was used for further processing to generate the reads needed for bacterial diversity statistics. On the QIIME2 pipeline, the adapters were trimmed using the trimmomatic tool (v 0.39) and the reads were demultiplexed using the ONT porechop tool (v0.2.4). The demultiplexed reads were checked for chimeric sequences using the VSEARCH—Qiime2 tool and the chimeric sequences found were filtered out using the UCHIIME tool. The reads were then aligned using MAFFT and taxonomy assigned using SILVA 132 database as the reference database. OTUs were generated from the reads using a 97% similarity threshold. The reads were normalized and rarefied to even sampling depths and both alpha and beta diversity calculated. Alpha diversity was calculated using the Shannon and Chao1 index in the phyloseq package in R^29^. Kruskal–Wallis test was applied to verify the alpha diversity differences between the treatments. To compute microbial beta diversity unweighted UniFrac analyses was performed and sample UniFrac distances visualized on PCoA plots. Multivariate analysis of beta diversity was verified using non parametric PERMANOVA using the Adonis package in R with 999 permutations (*P* < 0.05).

## Results

### Bacterial abundance composition

**Figure 1:**
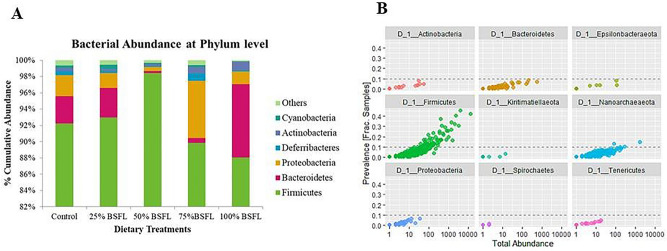
A stacked bar chart showing (**A**) Cumulative taxonomic composition at Phylum level; and (**B**) Prevalence of all observed phyla taxa in gut profiles of layers fed black soldier fly larvae feeds.

**Figure 2:**
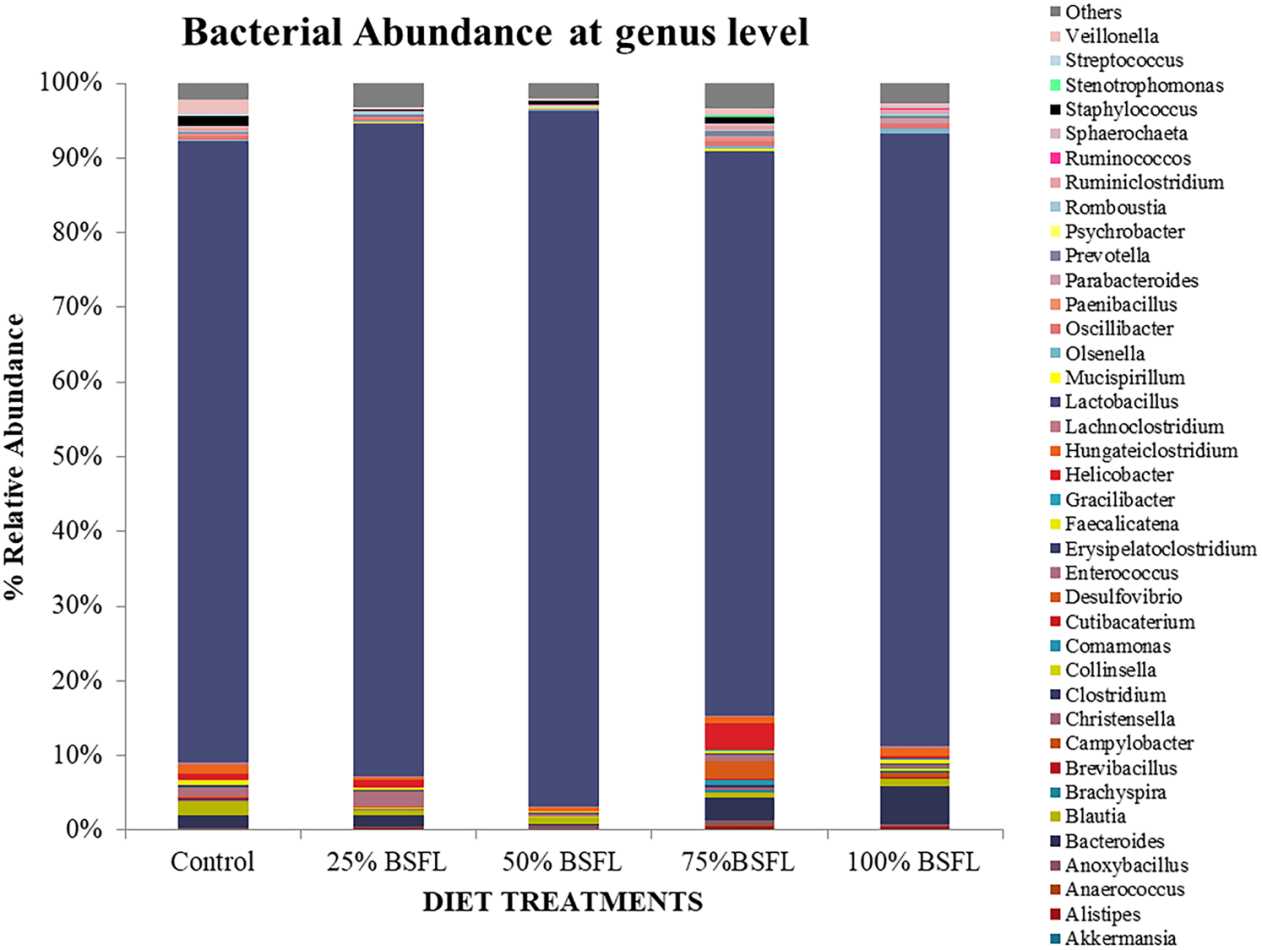
Cumulative abundance composition of bacteria Operational Taxonomic Units identified at the genus level across the different dietary treatments formulated from black soldier fly larvae–based feeds.

About 400,064 reads were generated from which 338,087 reads passed quality filtering and were classified with an average of 15,500 reads per treatment. The most abundant and prevalent phyla observed across all the treatment groups included Firmcutes (90%) followed by Bacteroidetes (7%) and lesser portions of Proteobacteria (2%) and Actinobacteria (1%) (Fig. 1A,B)*.* We characterized 38 main genera with a relative abundance of 0.5% and genera with a relative abundance of < 0.5% were collapsed into ‘others’ (Fig. 2). Cumulative relative abundance at the genus level showed that *Lactobacillus* (93%) which is associated with the phylum Firmicutes was the most predominant genus across all the treatment groups, followed by *Bacteroides* (4%), which is associated with the phylum Bacteroidetes (Fig. 2)*.* There was a significant increase in the beneficial bacteria such *as Lactobacillus, Bacteroides, Blautia* and *Enterococcus* (Fig. S1). This increase was observed as the inclusion level of BSF larvae meal in the diet increased. In T5 which had 100% BSF, the levels of beneficial bacteria increased considerably (Fig. S1A). However, some reads corresponding to possible clinical pathogenic bacteria such as *Campylobacter, Clostridia, Staphylococcus* and *Streptococcus* were present mostly in T4 and T5 though at very low amounts of less than 1% which are permissible according to the feed safety guidelines by Kenya Bureau of Standards (KEBS)**.** In T3, the clinical pathogens were minimal thus the beneficial bacteria were in surplus (Fig. 2).

**Figure 3:**
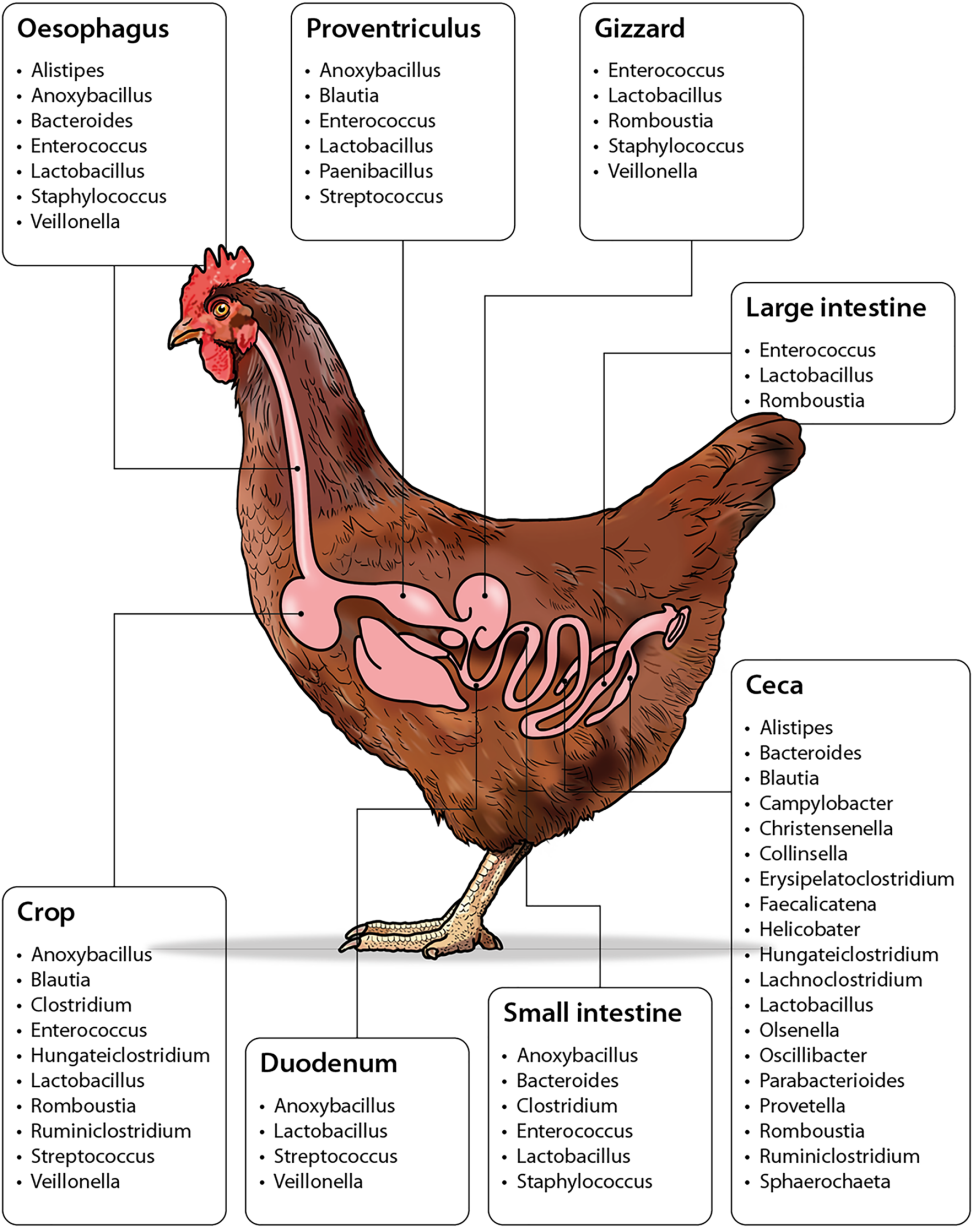
Bacteria genera identified across the entire gut segments of layer chicken fed on black soldier fly larvae feeds.

*Lactobacillus* species was the most abundant bacteria that dominated all parts of the chicken gut including the esophagus, crop, proventriculus, gizzard, ileum, large intestines, and the ceca. The ceca was the most diverse segment and it was dominated by the phylum Firmicutes*,* whereas the small intestines and large intestines had the least diversity and were colonized by Proteobacteria (Figs. 3, S3).

### Alpha and beta diversity

**Figure 4:**
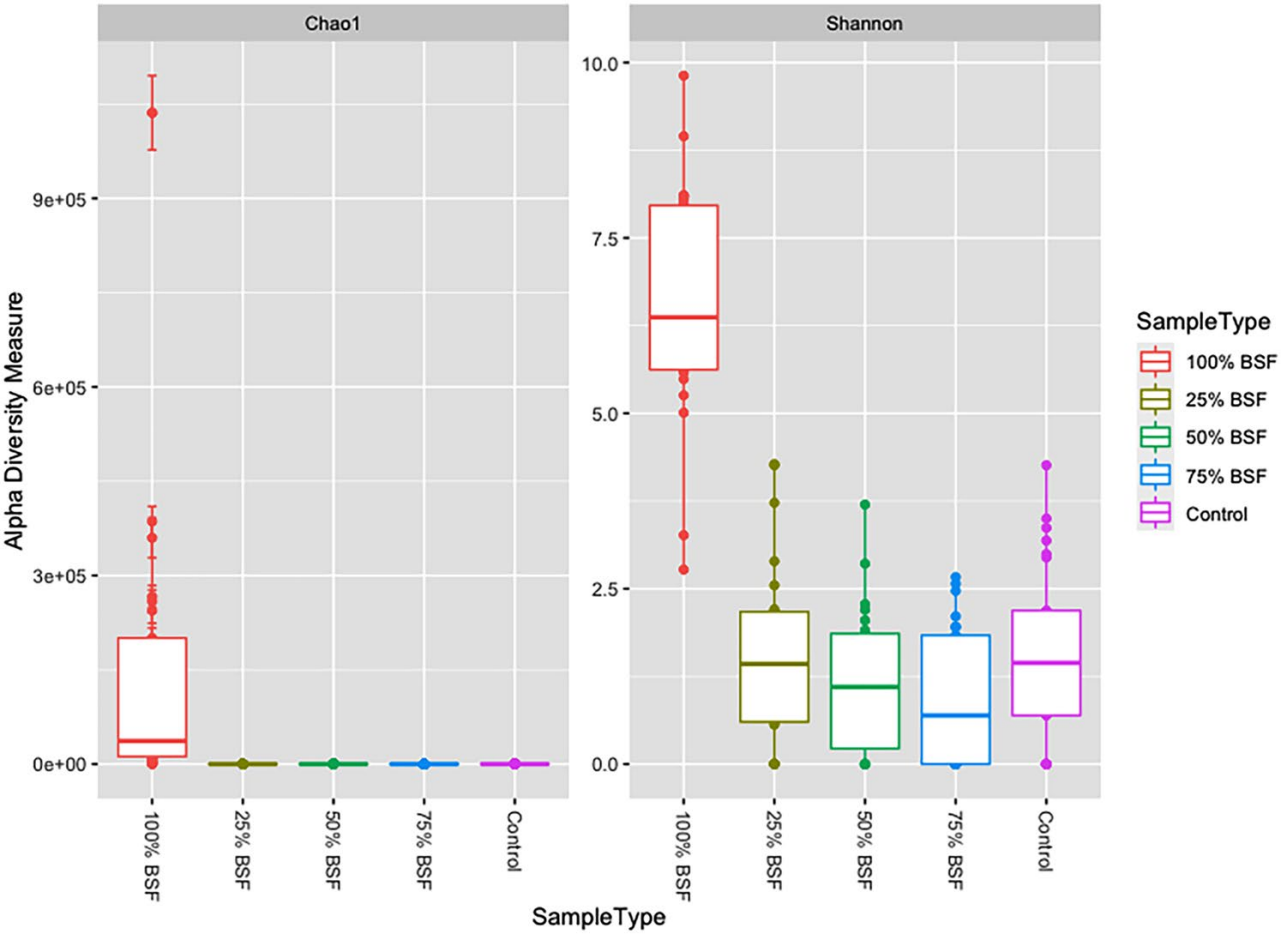
Alpha diversity measure using Shannon index and Chao1 richness index showing samples according to dietary treatments.

Alpha diversity was assessed using Shannon index showed that diet T5 which had 100% BSF was the most diverse group while T3 and T4 which had 50 and 70% BSF, respectively, had the least diversity (Fig. 4). Chao1 richness index showed that T5 (100% BSF) had the highest species richness and the most abundant read count with over 600 species identified.

**Figure 5:**
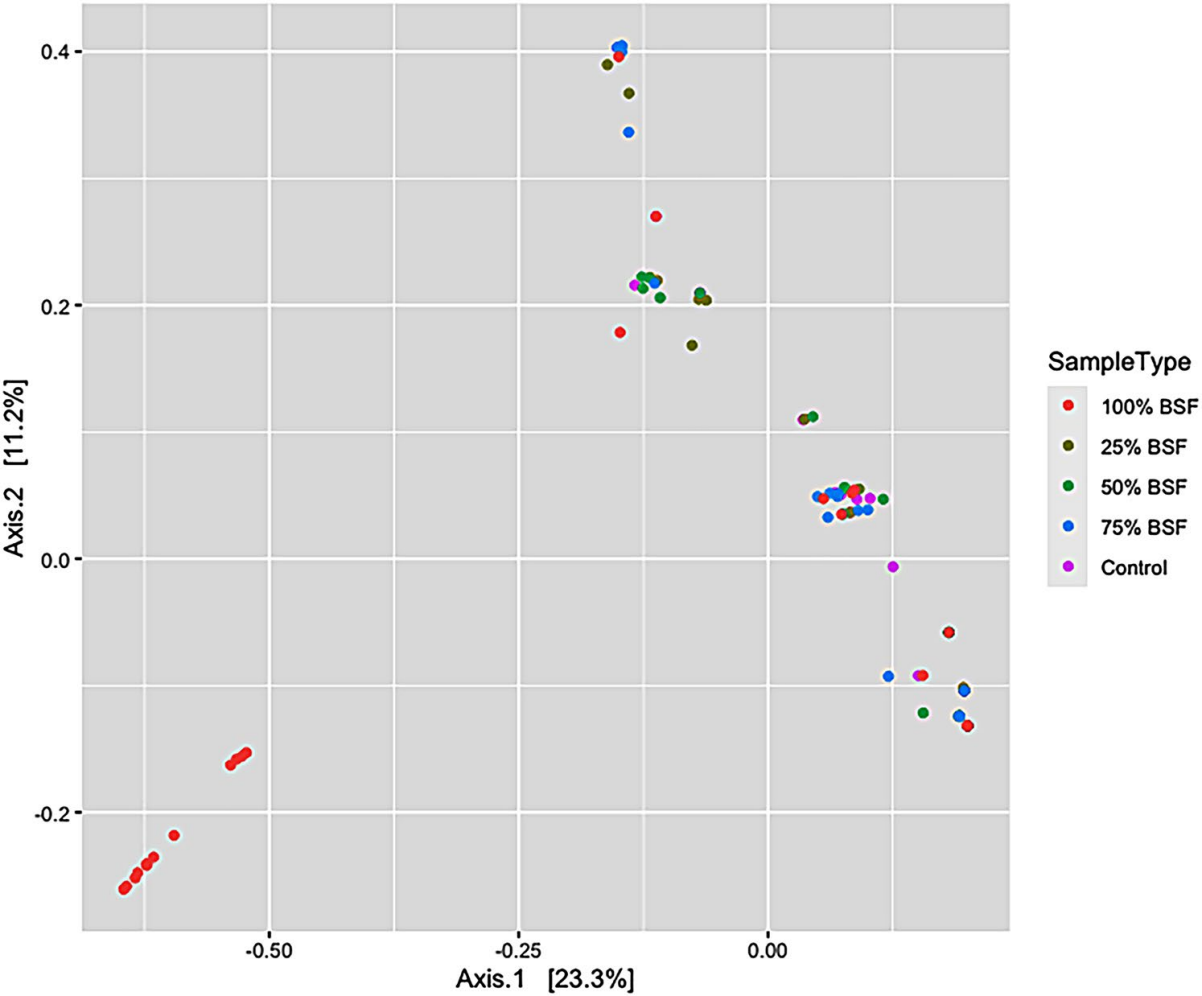
Beta diversity PCoA plot based on unweighted unifrac distance dissimilarity method between the different dietary treatments. [PERMANOVA] *R*-Squared: 0.11509; *p* value > 0.05.

Beta diversity which was calculated using the unweighted unifrac distance method indicated that diet treatment did not adversely affect the diversity of the microbial communities which all appeared to cluster together. Only slight differences were observed in the microbial composition and abundance between the different dietary treatments (Fig. 5).

**Figure 6:**
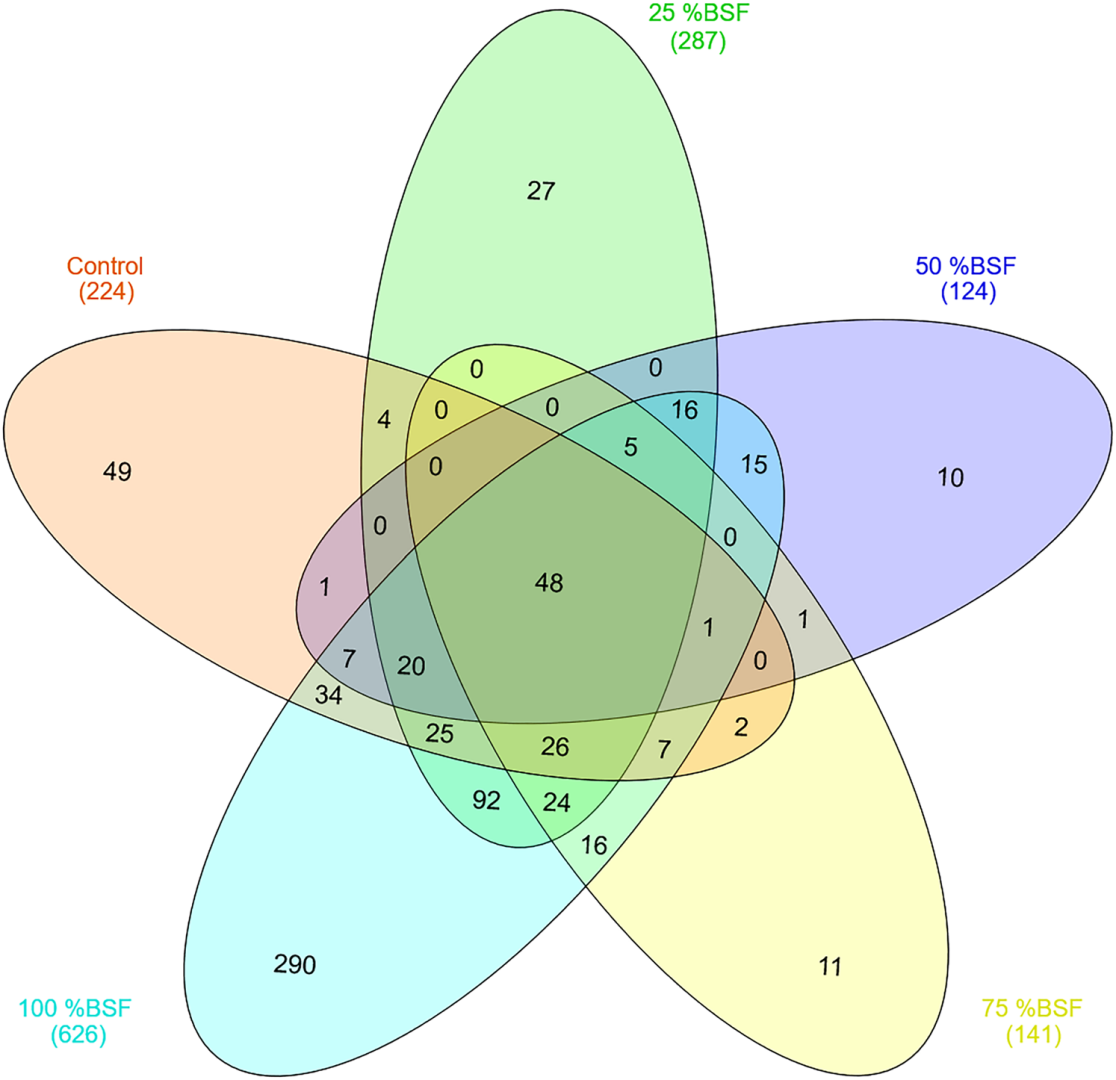
Venn diagram showing the identified shared and unique Operational Taxonomic Units between treatments with T5 having the most identified unique species.

A Venn diagram showed that 48 bacteria genera were common across all the five treatments. The 100% BSF treatment (T5) had the most unique genera totaling to 290 (Fig. 6).

## Discussion

Understanding the modulation of the gut microbial community by factors such as diet is very crucial as an essential component to chicken health^20,30^. Black soldier fly larvae (BSFL) meal is a promising and sustainable alternative to the expensive fishmeal in Layer chicken feed and a study done by^26^ confirms that replacing fishmeal with BSFL does not comprise the overall growth and economic performance of the Layer chicken. This study aimed to analyze the effects of feeding black soldier fly (BSF) meal on the gut microbial composition of layer pullets using Oxford Nanopore 16S sequencing. Here we observed that the partial replacement of conventional fishmeal with BSFL based diets had a positive influence on the gut microbial communities of the layer pullets. This report is related to a previous study by^25^ which reported that inclusion of BSF based meal in laying hens diets did not have a negative effect on their nutrition and gut microbiota composition. High microbial richness and diversity was observed in diets that had high concentrations of BSF especially on the treatment diet which had 100% BSF inclusion. Several studies have recently reported that a high bacterial diversity tends to be associated with a healthy gastrointestinal tract which improves the overall health status of poultry^25,31^. Therefore, the layer chicken fed BSFL in our study may have a healthier gut microbial community since the species richness and diversity was higher than the standard conventional fishmeal diet.

We identified across all treatment diets that the most predominant bacteria phyla included Firmicutes (90%), Bacteroidetes (7%), Proteobacteria (2%) and Actinobacteria (1%). Lesser portions of Cyanobacteria, Deferribacteres, Spirochaetes, Tenericutes and Verrucomicrobia were also observed. Our findings support previous reports showing Firmicutes, Bacteroidetes, Proteobacteria and Actinobacteria to be among the dominant phyla across all parts of the chicken gastrointestinal tract^19,21,32^. The phylum Firmicutes represents the most beneficial bacteria classes that are important in both animals and humans including Lactobacillales*,* Bacillales*,* Clostridia and Veillonellales^33^*.* The most abundant genera observed across all the dietary treatments were mainly *Lactobacillus, Enterococcus* and *Blautia* which are associated with the phylum Firmicutes and *Bacteroides* that are associated with the phylum Bacteroidetes*.* However, diets with BSF meal inclusion supported the same microbial communities as the standard fishmeal diet at both phylum and genus level with only a few taxa changing in abundance. A significant increase in the lactic acid bacteria (*Lactobacillus, Enterococcus* and *Bacteroidetes*) was observed in the diets that had a higher concentration of BSF, thus implying black soldier fly larvae-based feeds (BSFLF) might have the potential to boost the probiotic activities in the gut of the chicken. Lactic acid bacteria (LABs) have been known to produce bacteriocins which are said to inhibit the pathogenic bacteria in the gut thus they may be used as indicators of a healthy gut^31^.

Across the entire gut, the highest diversity of bacteria was observed in the ceca, while the lowest diversity was recorded in the esophagus, proventiculus and small intestines. This low density could be because of the short passage time in these parts and the dilution of the digesta in the small intestines^22^. *Lactobacillus*, of the family Lactobacillaceae, was observed as the most dominant taxa in all the dietary treatments and all the gut parts with the highest read counts being recorded in the crop and the cecum. This family of bacteria are beneficial commensals which have been studied extensively in the food and medicine industries concerning both humans and animals^34^. *Lactobacillus* species being an intrinsic component of the intestinal microbiota are now being acknowledged as the most efficacious probiotic candidates. These lactic acid producing bacteria have been reported to have various health benefits in human health some which include stimulation of immune responses, anti- cancer activity, prevention and treatment of inflammatory diseases^35^, lactose intolerance alleviation, antimicrobial activity against resistant pathogens and respiratory viral infections^36^. *Lactobacillus* species have been identified to produce lactic and acetic acids which help in lowering the pH value in the gut and competing with potential pathogens for nutrients through direct competitive interactions thus suppressing the colonizing potential of some major pathogens^34^. In addition, *Lactobacillus* have been used as alternatives to antibiotic growth promoters in poultry and as feed supplements to improve growth performance^23^.This may reduce the administration of antibiotics in feed in poultry production systems. Antibiotics have been extensively used as growth promoting agents in poultry production and this has prompted them to be under intense scrutiny due to their adverse effects such as increase in emergence of resistant pathogenic bacteria and risk of zoonotic transfer of resistant pathogens to humans^37^. *Lactobacillus* is being considered as a safe alternative to antibiotics because of its mode of action as a probiotic in animals. In poultry health, probiotics have been identified to have beneficial effects on intestinal histological changes, growth performance, immunomodulation as well as improving the sensory characteristics of poultry meat^38^. Alayande et al.^39^ reported in his studies that *Lactobacillus* stimulates the growth of beneficial microbes in animals preventing colonization of enteric pathogens through their ability to produce antimicrobial substances. This results are similar to a study by^40^ who observed that the use of *Lactobacillus* as a probiotic in pig production improved the growth performance with increased growth rates and feed conversion rates. These various studies show that *Lactobacillus* as a probiotic has a great potential as an alternative to antibiotic agents in poultry production. *Bacteroides*, *Enterococcus* and *Blautia* were also identified as abundant beneficial bacteria from our samples and their role in the degradation of polysaccharides to produce short chain fatty acids (SCFAs) such as acetic acid, butyrate, propionate and lactate has been well documented^33^. These SCFAs are known to reduce undesirable and detrimental bacteria by inhibiting acid sensitive pathogens and promoting mucosal growth by stimulating gut epithelial cell proliferation. They also act as a source of energy and stabilize glucose levels in poultry^41^.

Notably, some pathogenic bacteria of the genus *Campylobacter* were identified including species of *Campylobacter jejuni*, *Campylobacter lari, Campylobacter coli* and *Campylobacter upsaliensis*. *Campylobacter* bacteria were observed in the ceca of layer chicken fed on diet 4 (T4) and 5 (T5) which had 75% and 100% BSF, respectively, suggesting that black soldier fly may act as a mechanical vector transmitting the pathogenic strains to the chicken through the feed in high concentrations. However, the abundance of the detected bacteria was among the permissible levels as set by the Standards Projects Committee, developed by^42^ on use of insects for feed. From a recent study conducted by^18^ on microbial dynamics of the black soldier fly reared on different organic substrates, *Campylobacter* bacteria was identified as one of the prevalent species in the gut of unprocessed black soldier flies. *Campylobacter* are associated with the phylum Proteobacteria and they are the main pathogenic bacteria identified in poultry which in high amounts can cause campylobacteriosis in humans^43^. As a result, we recommend the use of safe and sterile substrates when rearing the BSF for feed to eliminate such contaminants that may have an impact on the advantages of using BSF larvae in poultry production.

## Conclusions

Our study was able to unravel various bacteria of importance in poultry health and the data demonstrated that black soldier fly larvae meal is a valid alternative animal protein to replace fishmeal in poultry feeds. These beneficial bacteria could be exploited further in the poultry industry to produce probiotics and prebiotics to improve the poultry health and nutrition. However, there were traces of harmful pathogenic bacteria which may have been picked from the rearing substrates. Therefore, the choice of organic substrate used in rearing BSFL is crucial to minimize vertical transmission of pathogens which could pose health risks to poultry and consumers. Further processing techniques post-harvest of the larvae from the substrate should ensure that all pathogenic agents are completely eliminated to limit their transfer into the feed value chain. Insect-based meal inclusion in poultry diet should be considered following establishment of safety measures such as better processing and storage techniques, sterilization of organic substrates before rearing of the BSF and proper diet formulation methods to eliminate microbial contaminants and produce feeds that ensure microbial safety of the poultry populations.

## Supplementary Information

Supplementary Information.

## Data Availability

The datasets generated during and/or analyzed during the current study are available from the corresponding author on reasonable request.

## References

[1] FAO. *The state of food insecurity in the World*. http://www.fao.org/3/a-i4646e.pdf (2015).

[2] Fasolato L, Cardazzo B, Carraro L, Fontana F, Novelli E, Balzan S (2018). “Edible processed insects from e-commerce: Food safety with a focus on the Bacillus cereus group. Food Microbiol..

[3] Van Huis, A. *et al.* Edible inseFood and agriculture organization of the United Nationcts. *Future Prospects Food Feed Secur* **171** (2013).

[4] Cullere M (2016). Black soldier fly as dietary protein source for broiler quails: Apparent digestibility, excreta microbial load, feed choice, performance, carcass and meat traits. Animal.

[5] Józefiak D (2016). Insects—A natural nutrient source for poultry—A review. Ann. Anim. Sci..

[6] Martens SD, Tiemann TT, Bindelle J, Peters M, Lascano CE (2012). Alternative plant protein sources for pigs and chickens in the tropics—Nutritional value and constraints: A review. J. Agric. Rural Dev. Trop. Subtrop..

[7] Frempong NS, Nortey TNN, Paulk C, Stark CR (2019). Evaluating the Effect of replacing fish meal in broiler diets with either Soybean meal or poultry by-product Meal on Broiler Performance and total feed cost per kilogram of gain. J. Appl. Poult. Res..

[8] Leiber F (2015). Replacement of soybean cake by *Hermetia illucens* meal in diets for layers. J. Insects Food Feed.

[9] De Souza-Vilela J, Andrew NR, Ruhnke I (2019). Insect protein in animal nutrition. Anim. Prod. Sci..

[10] Onsongo VO (2018). Insects for income generation through animal feed: Effect of dietary replacement of soybean and fish meal with black soldier fly meal on broiler growth and economic performance. J. Econ. Entomol..

[11] Wolfman L, Sachs BA (2013). Is insect protein a sustainable alternative to soy and fishmeal in poultry feed?. J. Chem. Inf. Model..

[12] Diclaro, J. W. & Kaufman, P. E. Black soldier fly *Hermetia illucens* Linnaeus (Insecta: Diptera: Stratiomyidae). *EENY 461* (2012).

[13] Müller A, Wolf D, Gutzeit HO (2017). The black soldier fly, *Hermetia illucens*—A promising source for sustainable production of proteins, lipids and bioactive substances. Z Naturforsch. Sect. C J. Biosci..

[14] Wang Y-S, Shelomi M (2017). Review of black soldier fly (*Hermetia illucens*) as animal feed and human food. Foods.

[15] Mazza L (2018). Efficient co-conversion process of chicken manure into protein feed and organic fertilizer by *Hermetia illucens* L. (Diptera: Stratiomyidae) larvae and functional bacteria. J. Environ. Manag..

[16] De Smet J, Wynants E, Cos P, Van Campenhout L (2018). Microbial community dynamics during rearing of black soldier fly larvae (*Hermetia illucens*) and impact on exploitation potential. Appl. Environ. Microbiol..

[17] Khamis FM (2020). Insights in the global genetics and gut microbiome of black soldier fly, *Hermetia illucens*: Implications for animal feed safety control. Front. Microbiol..

[18] Tanga CM (2021). Organic waste substrates induce important shifts in gut microbiota of black soldier fly (*Hermetia illucens* L.): Coexistence of conserved, variable, and potential pathogenic microbes. Front. Microbiol..

[19] Oakley BB (2014). The chicken gastrointestinal microbiome. FEMS Microbiol. Lett..

[20] Kers JG, Velkers FC, Fischer EAJ, Hermes GDA, Stegeman JA, Smidt H (2018). Host and environmental factors affecting the intestinal microbiota in chickens. Front. Microbiol..

[21] Kohl KD (2012). Diversity and function of the avian gut microbiota. J. Comp. Physiol. B Biochem. Syst. Environ. Physiol..

[22] Yeoman CJ, Chia N, Jeraldo P, Sipos M, Goldenfeld ND, White BA (2012). The microbiome of the chicken gastrointestinal tract. Anim. Health Res. Rev..

[23] Torok VA (2011). Identification and characterization of potential performance-related gut microbiotas in broiler chickens across various feeding trials. Appl. Environ. Microbiol..

[24] Boccazzi IV (2017). A survey of the mycobiota associated with larvae of the black soldier fly (*Hermetia illucens*) reared for feed production. PLoS ONE.

[25] Coretti L (2017). Insect-based diet, a promising nutritional source, modulates gut microbiota composition and SCFAs production in laying hens. Sci. Rep..

[26] Sumbule EK (2021). Cost-effectiveness of black soldier fly larvae meal as substitute of fishmeal in diets for layer chicks and growers. Sustainability.

[27] Fass. *Guide for the Care and Use of Agricultural Animals in Research and Teaching*, no. January (2010).

[28] Bolyen E (2019). Reproducible, interactive, scalable and extensible microbiome data science using QIIME 2. Nat. Biotechnol..

[29] McMurdie PJ, Holmes S (2013). “Phyloseq: An R package for reproducible interactive analysis and graphics of microbiome census data. PLoS ONE.

[30] Lin Q (2018). The chicken gut metagenome and the modulatory effects of plant-derived benzylisoquinoline alkaloids. Microbiome.

[31] Huyben D, Vidaković A, Werner Hallgren S, Langeland M (2019). High-throughput sequencing of gut microbiota in rainbow trout (Oncorhynchus mykiss) fed larval and pre-pupae stages of black soldier fly (*Hermetia illucens*). Aquaculture.

[32] Shang Y, Kumar S, Oakley B, Kim WK (2018). chicken gut microbiota: Importance and detection technology. Front. Vet. Sci..

[33] Rychlik I (2020). Composition and function of chicken gut microbiota. Animals.

[34] Yan W, Sun C, Yuan J, Yang N (2017). Gut metagenomic analysis reveals prominent roles of Lactobacillus and cecal microbiota in chicken feed efficiency. Sci. Rep..

[35] Toshimitsu T, Ozaki S, Mochizuki J, Furuichi K, Asami Y (2017). Effects of Lactobacillus plantarum strain OLL2712 culture conditions on the anti-inflammatory activities for murine immune cells and obese and type 2 diabetic mice. Appl. Environ. Microbiol..

[36] Ayeni FA (2009). Inhibition of uropathogens by lactic acid bacteria isolated from dairy foods and cow’s intestine in western Nigeria. Arch. Microbiol..

[37] Vieco-Saiz N (2019). Benefits and inputs from lactic acid bacteria and their bacteriocins as alternatives to antibiotic growth promoters during food-animal production. Front. Microbiol..

[38] Kabir SML (2009). The role of probiotics in the poultry industry. Int. J. Mol. Sci..

[39] Alayande KA, Aiyegoro OA, Ateba CN (2020). Probiotics in animal husbandry: Applicability and associated risk factors. Sustain..

[40] Dowarah R, Verma AK, Agarwal N (2017). The use of Lactobacillus as an alternative of antibiotic growth promoters in pigs: A review. Anim. Nutr..

[41] Borrelli L (2017). Insect-based diet, a promising nutritional source, modulates gut microbiota composition and SCFAs production in laying hens. Sci. Rep..

[42] Kenya Bureau of Standards. *DRAFT KENYA STANDARD Edible Insects Part 2: Products Containing Specification Edible Insects* (2020).

[43] Fravalo P, Lahaye L, Thibodeau A, Arsenault J, Letellier A, Yergeau É (2015). Chicken caecal microbiome modifications induced by *Campylobacter jejuni* colonization and by a non-antibiotic feed additive. PLoS ONE.

