# Supplementary material for: Gut microbiota shift in layer pullets fed on black soldier fly larvae-based feeds towards enhancing healthy gut microbial community
Source: Sci Rep. 2022 Oct 6;12:16714. doi: 10.1038/s41598-022-20736-0 (PMC9537291; doi:10.1038/s41598-022-20736-0)
Supplement: Supplementary file 1 — Supplementary Information. [file 41598_2022_20736_MOESM1_ESM.docx]

**Supplementary Data**

**(A)**

**(B)**

Fig S1: bar plot showing the increase in abundance of the most abundance beneficial bacteria genus (A) and other beneficial bacteria identified (B) across the diet treatments with increase in black soldier fly larvae meal inclusion.


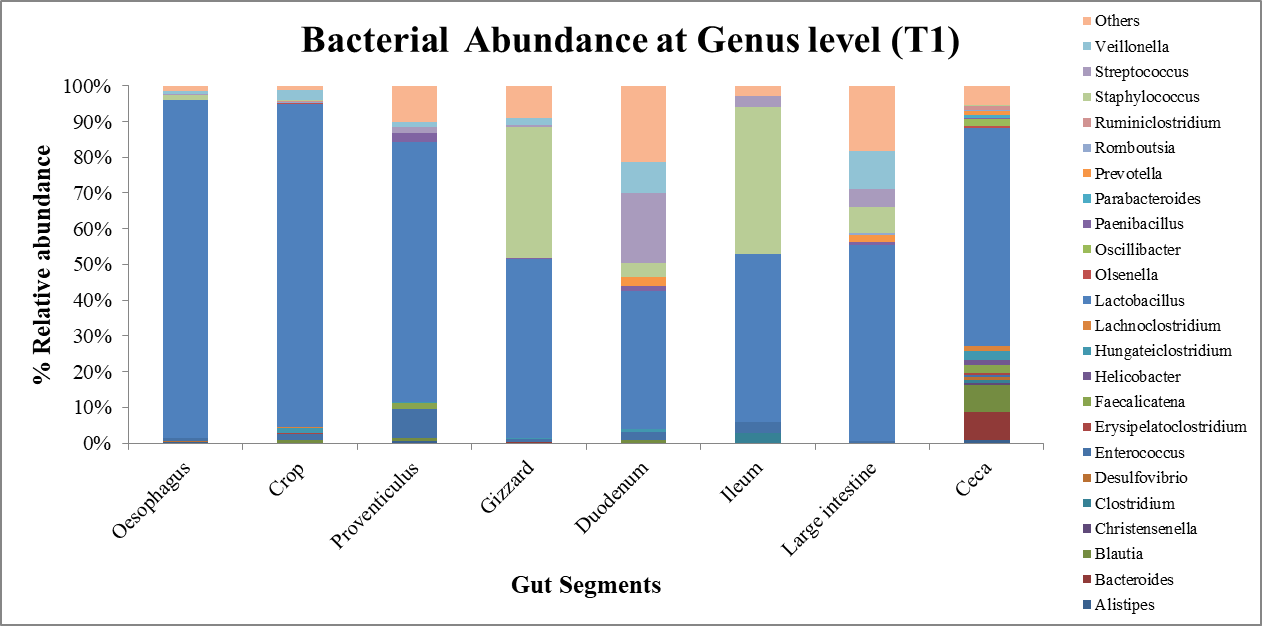


**(A)**


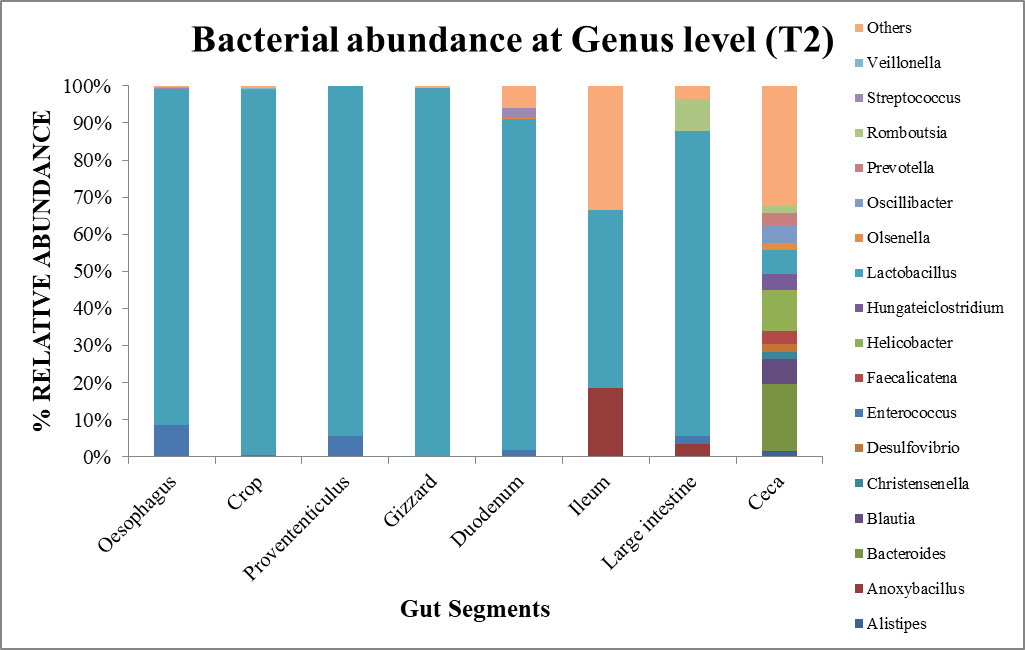


**(B)**

**
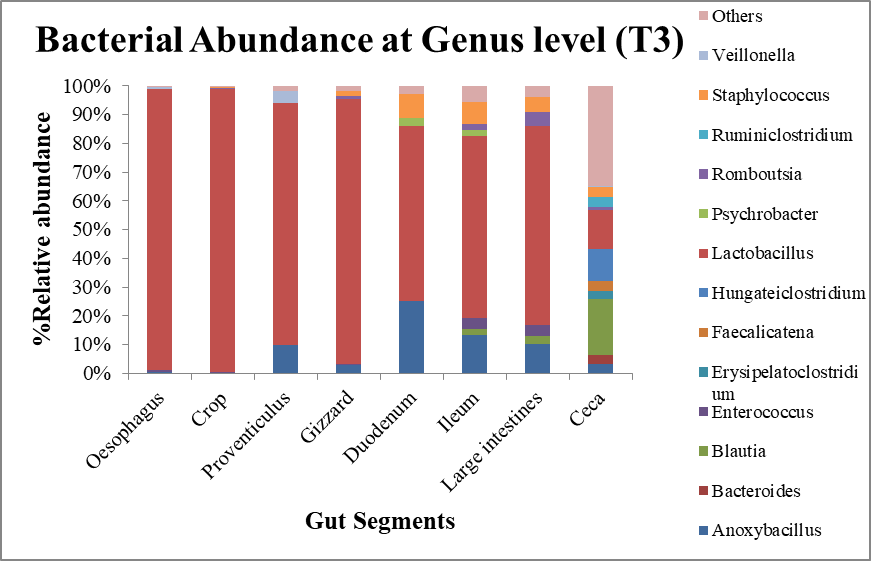
**

**(C)**

**
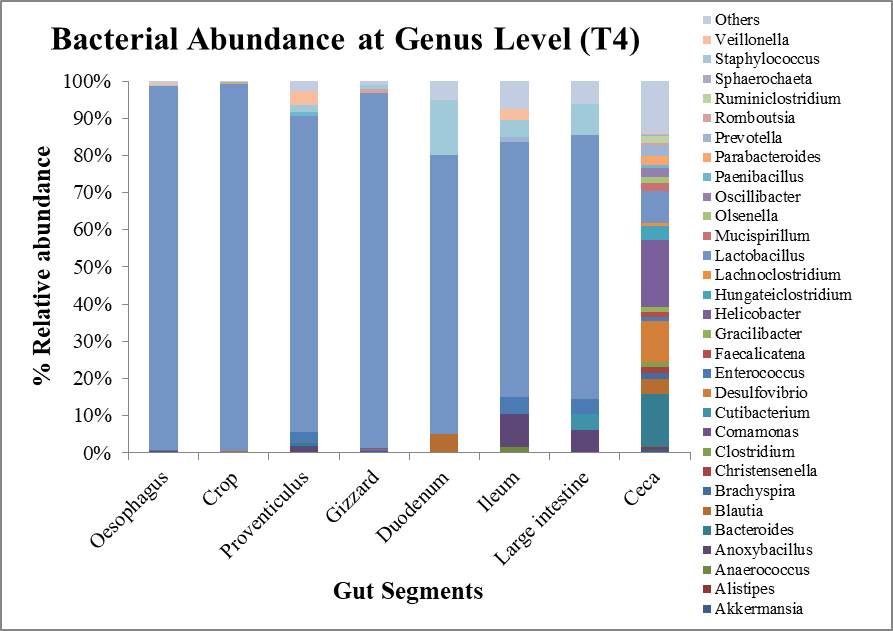
**

**(D)**

**
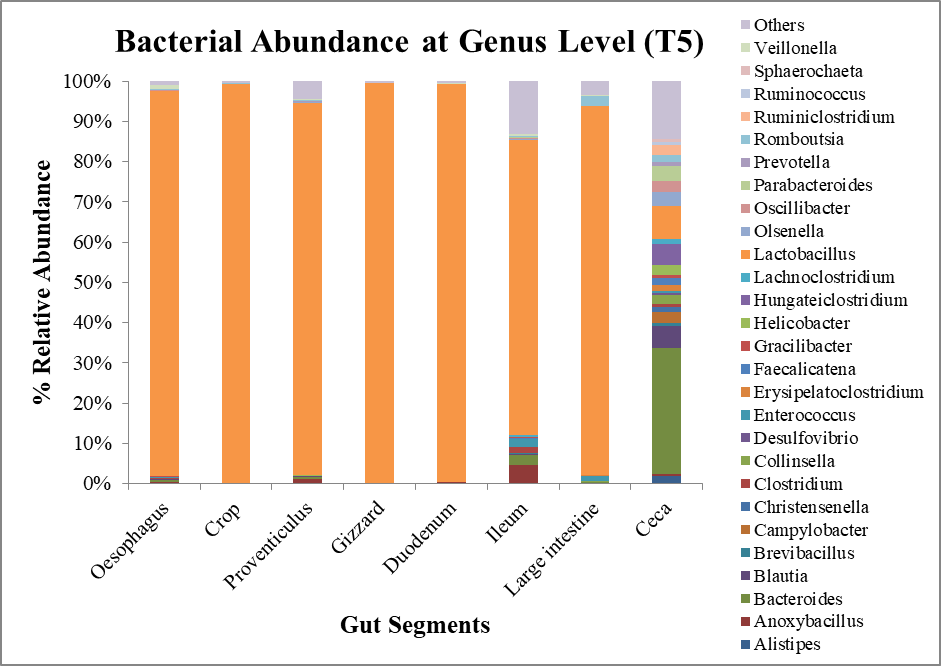
**

**(E)**

**Fig S2:** Bacterial microbiota cumulative profiles identified across the eight gut segments sampled from the gut of layer chicken fed BSF-based feeds with different inclusion levels **(A)** BSF inclusion level at 0%; **(B)** BSF inclusion level at 25%; **(C)** BSF inclusion level at 50%; **(D)** BSF inclusion level at 75%; **(E)** BSF inclusion level at 100%.

Table S1: Layer vaccination program from day 1 up to 19 weeks

| DAY | VACCINE | METHOD |
| --- | --- | --- |
| 1 (done in the hatchery) | Mareks + IBD- Vaxxitek  NCD + IB Live (Vitabron) | Intramuscular injection  Spray (done in the hatchery) |
| 15-18 | NCD + IB Live | Eye drop/Drinking water |
| Week 6-8 | NCD killed or  NCD + IB Live  Fowl typhoid | Intramuscular injection  Drinking water  Intramuscular injection |
| Week 8-10 | Fowl pox  Fowl cholera | Wing stab  Subcutaneous injection |
| Week 12-14 | Fowl typhoid | Intramuscular injection |
| Week 16-19 | NCD + IB Live  Fowl cholera | Drinking water/Spray  Subcutaneous injection |
